# Capturing Community Living Experiences and Health of Korean Community Spinal Cord Injury Population: A Cross-Sectional Survey

**DOI:** 10.3390/ijerph22081222

**Published:** 2025-08-05

**Authors:** Boram Lee, Hyeong Jun Kim

**Affiliations:** 1 National Rehabilitation Center, Seoul 01022, Republic of Korea; 2 Korea Health Industry Development Institute, Cheongju-si 28159, Republic of Korea; pitt908@khidi.or.kr

**Keywords:** spinal cord injuries, community survey, disability, health, activity and participation

## Abstract

(1) Background: People living with spinal cord injury (SCI) face numerous challenges in their lives in terms of health conditions, everyday activity, and participation in society, which are not fully recognized. To address such issues, a community survey with 125 questions for people living with SCI was conducted and the response rates, population characteristics, health and functioning problems are reported. (2) Methods: The survey questionnaire comprised 125 questions on SCI characteristics, health conditions, activities, participation, and environmental and personal factors. The survey response rates were calculated, and demographics and health and functioning characteristics were analyzed. (3) Results: A total of 890 individuals responded to the survey. The median age of the participants was 48 years (interquartile range (IQR), 39–56), and 76% of the population were males. Paraplegia (60%) and complete injury (58%) were the most common injury type, and the cause was mostly traumatic (92%). More health problems and lower quality of life were more frequent with older age and in patients without paid work. (4) Conclusions: The Ko-InSCI study provides valuable information in terms of health needs and service gaps for people with SCI in the community.

## 1. Introduction

Spinal cord injury (SCI) is a high-cost and medically complex condition that poses a significant challenge to the global health system and society [1]. The scarcity of evidence regarding the living experiences and needs of people with SCI in Korea renders it more difficult to overcome its challenges [2]. Although a National Disability Survey is held every 3 years by the Ministry of Health and Welfare, people with SCI are not studied as a distinct population, but rather included in the depiction of a broader category of people with “physical disability” [3]. This lack of information on the lives, health, and functioning is a significant problem as it leads to lack of evidence-based disability-specific health management services, especially with the growing health care needs in the SCI population [2,3,4]. Although limited to certain contexts in other countries, studies investigating unmet needs for community dwelling people with SCI have been carried out by capturing environmental barriers, health conditions, life satisfaction, and expressed needs [4,5,6]. Participation of people with SCI in the community and environmental barriers affecting them were identified in previous studies, in which physical barriers, such as transportation and accessible infrastructures, and financial barriers were considered most influential [5,7,8]. In addition, multiple concurrent secondary health problems such as pain, spasticity, and bladder and bowel dysfunctions following SCI have been reported in numerous studies with findings showing lower perceived quality of life (QoL) associated with high rate of secondary health problems [6,9,10]. To address such unmet needs, efficiently facilitate participation in community and eventually enhance QoL, multiple aspects of their health, functioning, and barriers need to be systematically collected and analyzed in different contexts globally, as well as in Korea.

The lack of data on an international level is also described in the International Perspectives on SCI report and WHO’s World Report on Disability [1,11]. Rooting from previous reports, the Learning Health System for Spinal Cord Injury (LHS-SCI) initiative aimed at improving the lived experience of people with SCI by generating internationally comparable and relevant data through formulating an International SCI community survey [12]. It is designed to be repeated at 5-year intervals to capture ongoing real-time experiences of people with SCI. This community survey, which will be followed by many surveys in the future, will capture the lived experiences of SCI in different socio-cultural contexts, and provide opportunities to capture and learn from good practices by obtaining longitudinal information across different countries.

Stemming from the belief in the importance of generating internationally comparable data, Korea has actively joined the initiative and has participated as one of the 22 countries in the International Spinal Cord Injury (InSCI) community survey. In this study, we aimed at describing the Korean protocol of the InSCI survey (Ko-InSCI), reporting the participation rates and describing the characteristics of the participants, examining the health and functioning status of the participants through analyzing how they differ in terms of sociodemographic and lesion variables, and providing future implications for the next survey by highlighting the achievements and limitations of the protocol.

## 2. Materials and Methods

### 2.1. Study Protocol and Participants

The Ko-InSCI study was performed in one of the 22 countries that participated in the first InSCI study. Accordingly, the researchers followed the recommendations and instructions in the InSCI community survey protocol provided by the InSCI study center (Swiss Paraplegic Research, Notwil, Switzerland) [13]. A cross-sectional study using a common structured questionnaire was conducted between March 2017 and January 2018 in Korea.

The eligible study participants were adults aged ≥19 years living in a community in Korea with traumatic or non-traumatic (infections, vascular causes, toxic agents, or malignant tumors) SCI. The exclusion criteria included the patients (1) who were unable to understand and respond to the questions due to cognitive impairment or communication problems, (2) with congenital or neurodegenerative etiology of SCI, (3) having peripheral nerve damage such as Guillan–Barré syndrome, or (4) in their acute care or first inpatient rehabilitation program.

Owing to the lack of SCI-specific national registry in Korea, the target study population was estimated based on the 2014 National Disability Survey. According to the survey, a total of 1,373,737 people are estimated to have physical disability, in which 4.9% of the cases accounted to have SCI as its etiology, resulting in approximately 67,313 people with SCI [3].

The source population was based on patient records from the National Rehabilitation Center (NRC) and Korea Spinal Cord Injury Association (KSCIA) members. Although there may be concerns of generalizability since the population is only from two institutions, the two institutions distinctively have the largest database of people with SCI as a hospital and a representative organization. The national rehabilitation center is the sole national rehabilitation center affiliated with the Ministry of Health and Welfare in Korea, which is especially well known for its high quality and special SCI rehabilitation programs and services. Accordingly, they are visited by the majority of SCI patients and have the largest number of beds for SCI admissions, and hence the largest patient database in the country, enabling it to produce epidemiological data of traumatic and non-traumatic SCI in Korea [14,15]. And KSCIA, with its nationwide network, is the only authorized and largest representative organization that only includes people with identified SCI as its members. They are known as the representative stakeholder for the people with SCI in the nation and also regularly carry out living status surveys on their members. The contact database, which was constructed using both records, unveiled a source population of 6584 individuals with 6355 eligible patients who were consequently invited to participate in the study. The target sample size that is representative of the target population with a 95% confidence level and 3% margin of error was 1050 patients. All eligible individuals in the database were recruited considering possibility of high rate of duplications between the two databases and non-response rate of approximately 50% as reported by the former SwiSCI study [16].

### 2.2. Recruitment and Data Collection

A mixed-mode response alternative was implemented as recommended by a previous SwiSCI study [16], including both online and paper-and-pencil questionnaires, and face-to-face interviews when needed or requested. Moreover, a recurrent mixed-mode reminder was provided to optimize the survey response rate [16]. The initial contact with the respondents was established through context messages, which included an invitation, information on the survey, and a link to the online survey version. Information was provided regarding contact with the Korean study center if the participants had further questions or if other modes of response were preferred or needed. In addition, the KSCIA members received a paper version of the questionnaire from their regional centers. Completed questionnaires were collected from each regional center. Prior to the recruitment process, notifications in KSCIA and other consumer homepages were sent to raise awareness about the first international community survey on people with SCI to achieve a higher response rate. Moreover, reminders were sent five times for each eligible individual. In the first three times, context messages were sent in the survey with an online survey link, which was identical to that in the initial message. For those who did not respond, two telephone calls were made at one-month intervals. Those who did not respond after the fifth reminder were classified as having “no contact”.

### 2.3. Survey Questionnaire

The Ko-InSCI community survey used the translated version of the InSCI international module for data collection; it consisted of 125 questions concerning sociodemographic factors, SCI lesion characteristics, body functions and structures, activities and participation, environmental factors, personal factors, and appraisal of health and well-being. Translation was performed using the final version of the InSCI International Module, strictly following the method presented by Beaton et al. [17]. This was a 6-step process consisting of translation, synthesis, backward translation, expert committee review, testing of the pre-final version of the questionnaire, and submission of documents to the committee for appraisal of the adaptation process. The questionnaire involved a pilot test with 100 community-dwelling individuals with SCI conducted through face-to-face interviews. Tests and retests were performed at a 1-week interval, and the test–retest reliability and internal consistency of the translated survey were evaluated. No additional national modules were used in the Ko-InSCI community survey.

### 2.4. Variables/Measures

The InSCI data model was based on the International Classification of Functioning, Disability, and Health (ICF) framework in terms of the selection of relevant contents for data measurement [12]. The model included well-established and validated measures such as the Spinal Cord Independence Measure, SF-36 vitality and mental health domains, Spinal Cord Injury-Secondary Conditions Scale, Nottwil Environmental Factors Inventory Short Form, and WHOQOL-BREF quality of life. These measures were included in the data analysis, along with those for sociodemographic and lesion characteristics, to provide a full description of the lived experiences of people with SCI in the community.

### 2.5. Statistical Analyses

Statistical analyses were performed using R (version 4.1.2; The R Foundation for Statistical Computing, Vienna, Austria) and T&F software (version 4.0; YooJin Biosoft, Korea). Descriptive statistics were used to describe the demographic and injury variables in terms of frequency, percentiles, and medians with interquartile ranges. The SCIM-SR score, number of activities and participation problems, NEFI-S score, number of health problems, mental health score, vitality score, self-efficacy score, QoL, and living conditions were compared among the different subgroups. One-way ANOVA was used to compare the mean differences between groups before adjusting for covariates, and ANCOVA was used to show the adjusted mean differences and 95% confidence intervals. The analyses were performed by leaving out the missing data for each variable.

## 3. Results

### 3.1. Study Participation and Study Population Characteristics

**Figure 1:**
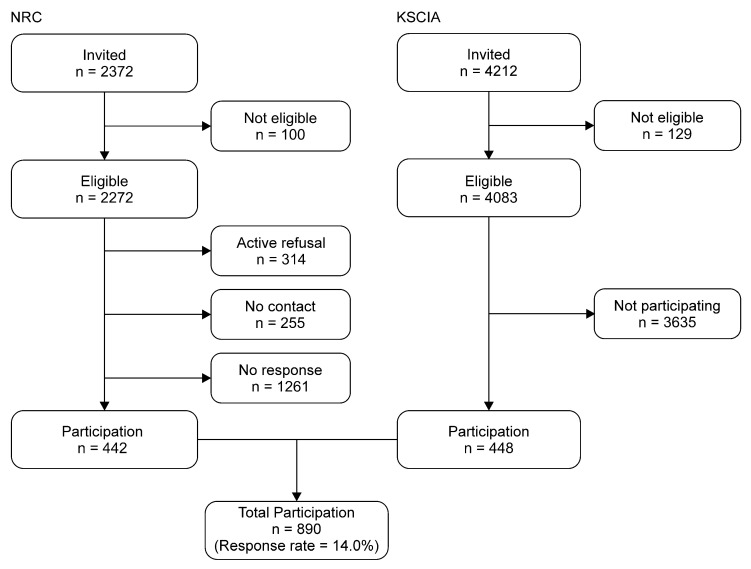
Flow diagram describing participation.

A total of 890 patients with SCI responded to the survey. The 14% survey response rate was calculated according to the definition of the American Association of Public Opinion Research (Figure 1) [18]. A total of 886 responses were self-administered and 4 were collected by face-to-face interviews. A total of 555 and 331 people responded to the paper-and-pencil and online questionnaires, respectively (Table 1). We were unable to compare the characteristics of the participants and non-participants because of the lack of detailed information regarding injury in terms of etiology, extent of lesion, precise lesion level, and duration since injury of the non-participants in the KSCIA database.

The sociodemographic and injury characteristics of the participants are presented in Table 2. Due to the self-answering nature of the questionnaire, some items were left unanswered by the participants, mostly due to unwillingness to answer or unavailable information, leaving missing responses for the items in Table 2: 3 responses missing for sex, 31 missing for age, 9 for partnership status, 75 for educational level, and 39 for household income. The median ages at the time of the survey and time of injury were 48 and 30 years, respectively. The median number of years since injury was 14.7 years. About 24% of the respondents were females which is similar to worldwide data [19], and 49% of the respondents reported that they were in a partnership. The median value of subjective social status was 4 out of 10 (1 being the lowest and 10 being the highest), and only 28% reported that they had paid work. The monthly household income showed the highest prevalence in the lowest income decile (34%), followed by the second income decile (21%); thus, more than half of the respondents corresponded to the lowest two levels. The frequency of respondents with higher income levels tended to decrease as the income levels increased. Many of the respondents had higher secondary education as their highest educational level (42%), and about 22% had a bachelor’s degree or higher level of education. Most participants lived with others, while 28% of participants reported living alone.

Paraplegia was more common than tetraplegia (60% and 40%, respectively). The rate of complete injury (58%) was higher than that of incomplete injury (42%). Complete paraplegia (40%) had the highest prevalence, followed by incomplete tetraplegia (22%), incomplete paraplegia (20%), and complete tetraplegia (18%). Compete paraplegia and complete tetraplegia were rated higher than the global average (28% and 10%, respectively), but incomplete paraplegia and tetraplegia were lower than the global rate (33% and 28%, respectively) [19]. The cause of SCI was mostly traumatic, accounting for 92% of all cases which was higher than the global average (81%) [19]. Traumatic injuries were primarily caused by traffic accidents (51%), falls (23%), and work-related accidents (12%). Accidents during sports (3.7%) or leisure activities (5.2%) accounted for only a small proportion of the accidents.

### 3.2. Activity and Participation

**Figure 2:**
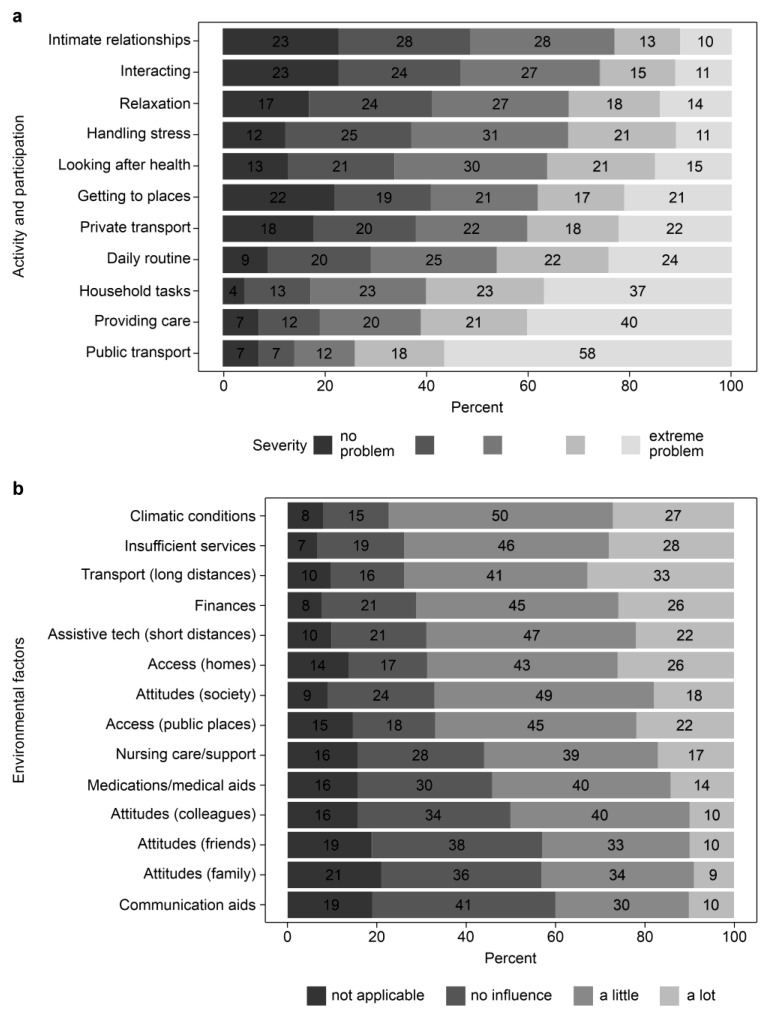
Activity, participation, and environmental factors. (**a**) Activity and participation; (**b**) Environmental factors.

Most Korean participants faced problems while using public transportation (86%). Similarly, they faced difficulties in using private transportation (62%) and getting to where they wanted to go (59%). Performing household tasks (83%), caring for others (80%), and carrying out daily routines (71%) were commonly reported challenges (Figure 2). The average number of moderate-to-extreme problems in activity and participation was nine out of eleven.

### 3.3. Independence in Activities of Daily Living

The mean SCIM-SR total score, which was calculated only from the items included in the survey, was 36 (possible range, 0–66). The highest rates of independence were reported in eating and drinking (77%), and grooming (57%). Additionally, a fair number of respondents reported that they could use the toilet either independently or with adaptive devices (43%). In terms of mobility, most respondents were able to transfer from bed to wheelchair with or without partial assistance, and 42% could sit independently in bed. To move around moderate distances (10–100 m), most respondents used manual or electric wheelchairs, and 24% required complete assistance.

### 3.4. Environmental Conditions

The average NEFI-S total score calculated from the given items was 37 (possible range, 1–56). Environmental factors negatively affected the lives of respondents in more than half of the cases. The most frequently reported factors were climate (77%), state services (74%), long transportation (74%), and financial hardships (71%). Moreover, short transportation, public access, and social attitudes were significant factors contributing to a difficult life. (69%, 67%, and 67%, respectively; Figure 2)

### 3.5. Health Conditions

**Figure 3:**
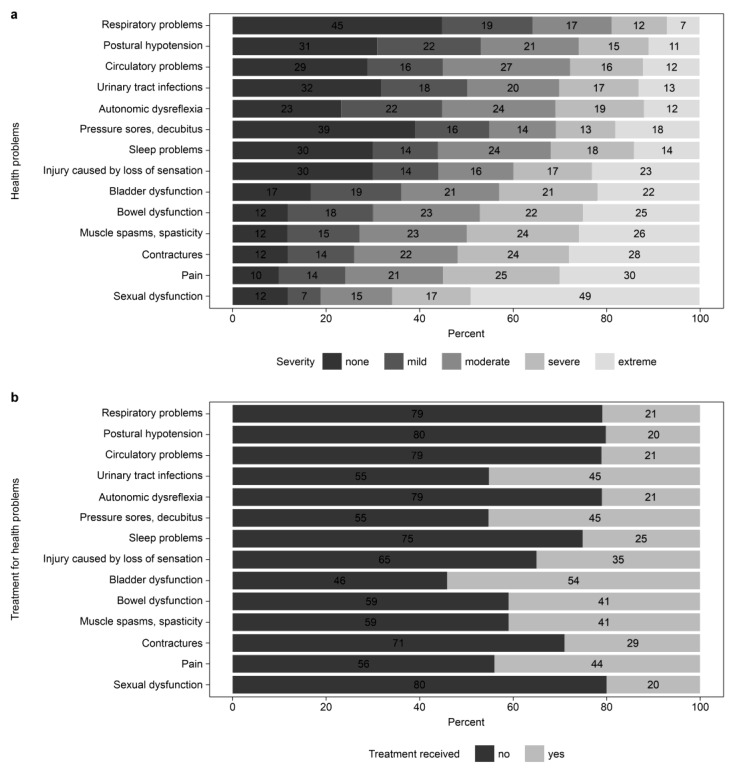
Health problems and treatment status. (**a**) Health problems; (**b**) Treatment for health problems.

In a recent study, high prevalence of comorbidity (93%) with a high average number of secondary health conditions (7.4) has been reported in the worldwide SCI population [10,20]. In our study, of the 14 secondary health conditions, the number of health conditions that caused any problem (mild-to-extreme in severity) was reported to have an average value of 10.1 (range, 0–14). The most reported complications were pain (90%), spasticity (88%), sexual dysfunction (88%), contracture (88%), bowel dysfunction (88%), and bladder dysfunction (83%), which were similar in order but showing higher prevalence to previous studies [20]. Of those health problems, the least treated complications were sexual dysfunction (20%), postural hypotension (20%), respiratory and circulatory problems (21%), and autonomic dysreflexia (21%) (Figure 3). Overall, a limited number of patients (20–54%) experiencing each of these problems received treatment. The average pain intensity for those who experienced pain was reported to be 5.46 out of 10, and 54% reported pain intensity higher than the average value. For overall rating of health, only 34% of the participants reported good-to-excellent health, while 25% of the participants stated that their health status was poor.

### 3.6. Mental Health and Vitality in Terms of Energy and Feeling

Some respondents were nervous (14%), felt down (17%), and depressed (17%) most of the time. Half of the respondents reported that they felt worn out to some extent, 38% felt tired commonly in their lives, and 50% were not feeling full of life. In terms of overall mental health, as shown in a previous study comparing 22 InSCI-participating countries, Korea was rated second to have the worst overall mental health status [21]. (Korea 58.1 vs. global average 66.3).

### 3.7. Personal Factors

In terms of self-efficacy, the respondents were most confident in maintaining social relationships (46%) and least confident in maintaining good health (22%). More than half of the participants (53%) were worried about their future and were unsure whether they would be able to achieve their dreams, hopes, and wishes (42%). They felt included in 43% of the cases, and 48% had a high sense of autonomy in their lives.

### 3.8. Subjective Quality of Life (Well-Being)

**Figure 4:**
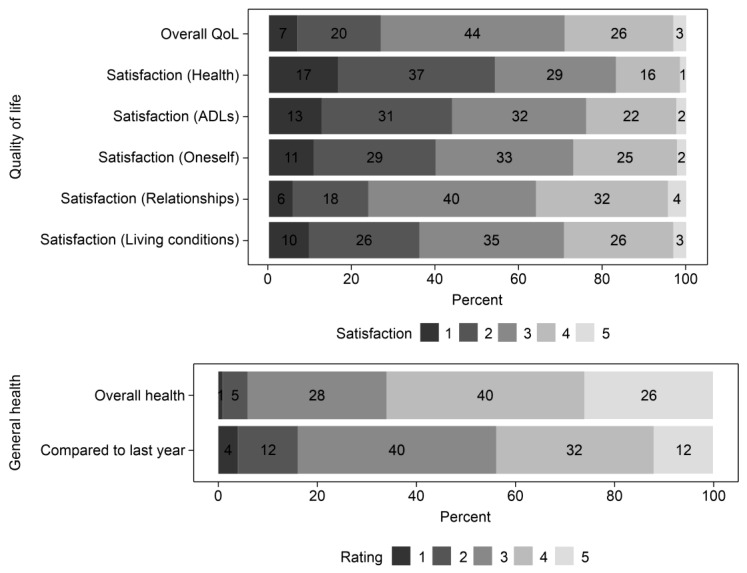
Quality of life and general health.

Many respondents reported that their overall quality of life was neither poor nor good (44%), with 29% reporting good or very good, and 27% reporting poor or very poor. Satisfaction with relationships had the highest rate (36%), whereas satisfaction with health had the lowest rate (17%). Accordingly, dissatisfaction with their health was high (54%), followed by dissatisfaction with their ability to perform daily living activities (44%) and with themselves (40%) (Figure 4).

### 3.9. Differences in Activity, Participation, Health Conditions, Quality of Life, and Environmental Factors According to the Sociodemographic and Lesion Characteristics

Patients with tetraplegia and those without paid work were associated with lower independence in performing activities of daily living, calculated as m-SCIM-SR scores. For activity and participation, participants with recent injuries, tetraplegia, unpaid work, and older age were associated with a higher number of problems. The NEFI-S score did not show significant differences among the groups but did show a tendency to increase with older age and decrease with longer injury duration. Older age subgroup and those who were in 6–15 years of duration of the injury were associated with higher number of health conditions. This was consistent with a study from Finland that showed more prevalent health conditions in the elderly [22]. Engagement in paid work was associated with fewer health problems. Additionally, aging population (≥61 years) and those without paid work were associated with lower overall QoL (Table 3 and Table 4).

## 4. Discussion

The Ko-InSCI study was the first systematically conducted nationwide community survey on the SCI population in Korea. Moreover, it was the first survey to attempt to capture the full range of lived experiences of individuals with SCI based on the ICF model. Although few previous studies reported the incidences and epidemiological trends of SCI concerning the lesion characteristics and etiology in Korea [23,24], no study comprehensively examined the socioeconomic status, activities of daily living, social participation, environmental and personal factors, working status, health problems, and QoL, while covering a significant proportion (1.3%) of the population. Being one of the participating countries, Korea has successfully recruited more than the minimal sample size of 200 participants as stated in the InSCI study protocol and has globally provided evidence that could be compared with the 21 participating countries [13,19].

Disability from SCI accounts for only a small proportion (2.5%) of the total population having disabilities in Korea, which is similar to the global proportion where SCI accounts for less than 0.1% of the total global population with disabilities [2,3]. Nonetheless, the information and study on the lives of patients with SCI could provide proxy measures on the effectiveness, equity, and accessibility of the health system since the social, physical, and psychological constraints faced by patients with SCI largely coincide with those of the rest of the population with disabilities. This study provided a great understanding of the lives of people with disabilities. It is the first study to measure the functioning status with the lesion characteristics and environmental factors for the individuals with disabilities in Korea.

The demographic characteristics of the population differed from those of recent previous studies in Korea, which showed an older age of onset and higher proportion of females and non-traumatic causes [23,24]. The differences could be attributed to the different data sources of the population, in which previous studies were limited to recent injuries, while our study included a wider range of injury durations with 14% of the participants exceeding 26 years. Our study population shows a better representation of the chronic SCI population living in the community than the previous studies.

In the study population, the self-reported socioeconomic status was surprisingly low; most participants positioned themselves in the lowest levels of the social status ladder, and most of the participants were in the three lowest income deciles. This could be attributed to the fact that only 28% of the respondents were engaged in paid work. The self-reported evidence can be supported by the recent investigation showing that the average yearly income gap between households of people with and without disabilities was almost double (2,267,000 vs. 4,317,000 Korean won, respectively) [25]. Accordingly, our study showed that financial hardship was one of the major environmental factors restricting participation, which implies the need for effective return to work program or pension that could sufficiently compensate for their low income.

Transportation was also a major problem affecting people’s lives, which is similar to previous studies from different countries [4,7,8]. Long-distance transportation was much more problematic, and most of the respondents had difficulty with public transportation. Accessible transportation itself is important, as traveling is an individual’s fundamental right [26]; however, there should be additional attention on its consequences as it may prevent individuals from receiving health services and education, and commuting to everyday work [11]. The need for prompt policy response to address public transportation accessibility is evident from the findings of this study.

Multimorbidity was common in the study population, and chronic pain, spasticity, and sexual and bowel dysfunctions were the most frequently reported secondary conditions, which are consistent with those reported in previous studies [10,20,27]. Korea is unique in that as reported in recent studies of 21 countries, it shows the highest average number of comorbidities, highest sum score of health condition severity, and one of the worst mental health statuses [20,21]. This might indicate the need for the redirection of health service delivery systems to meet these relevant health outcomes of the population. The national health management delivery system for people with disabilities has been established in 2018 in Korea, but it still struggles to set its service indicators on health outcomes of the disability population [28]; this needs to be promptly addressed for effective service development and delivery.

Moreover, the subjective health status reported by the Ko-InSCI population was relatively poor, which could be related to the finding of a high number of secondary health conditions with a high rate of non-treatment. In addition, in terms of self-efficacy, confidence in maintaining health was very low, and dissatisfaction with health was the highest among the QoL items included in the survey. These findings imply the existence of high unmet health care needs in the population, which are likely to result in poor life satisfaction and lower QoL in the population [9,29]. Even in countries with high health service coverage index including Korea, it has been reported that health systems frequently fail to effectively manage and monitor chronic conditions and disability [30]. The non-treated secondary conditions with high severity should be monitored in terms of service coverage by the health system in Korea and other countries as well, to ensure they are effectively managed. Moreover, additional health concerns regarding ageing and other chronic conditions not addressed in the questionnaire must be considered. For example, greater risk of developing metabolic syndrome in people with SCI is supported by substantial evidence recently; [31,32,33,34] and findings from our study and other previous studies implies that aging is associated with increased health conditions [22,35,36]. With such complex and changing health needs, health services that are both accessible and responsive to the SCI population, such as the primary care physician program for persons with disabilities in Korea, should be designed and located near their home. Since durations of 6–15 years and aging were associated with increased health problems, health examination programs 10 years after SCI onset and in the later ages should enable earlier screening for the SCI-specific secondary health conditions such as bowel and bladder complications, autonomic and cardiopulmonary functions, and metabolic diseases.

The study was not without limitations. The recruitment strategy included a possible selection bias because the study population included only one specialized rehabilitation center and one organization of persons with disabilities, which may not fully represent the entire target population of people with SCI in terms of geographical distribution, social participation, and relationships. Further, the rather low response rate of 14% may have been caused by the extensively detailed questionnaire and failure to motivate people to participate without providing any compensation. As response rate is defined as the percentage of those who participated of the total eligible persons, those who could not be contacted and not responded to the contacts and reminders were all classified as non-response. They may have turned out to be non-eligible once contacted but as they were unable to be identified, it adds to the high non-response rate. The low response rate of InSCI from other countries, as well, has also been considered in the previous literature, and it was identified that the low contact rate was again thought to be the reason [19]. In the current study, the inability to verify up-to-date contact information in the patient database may be the reason for the high non-response rate. Moreover, people who did not participate may have worse living experiences and present with severe mental or physical disabilities, which may have introduced potential participation and non-response bias leading to less representative survey results. These factors need to be considered in interpreting the results of the study. To accommodate for such possible bias, non-responder analysis with information on several variables of the non-responders should be performed as described by Fekete et al. [16,37]. However, we were unable to acquire further information for those participants from KSCIA other than age, sex, and etiology because they did not have a fully defined database of the members. Alternatively, we compared the key characteristics with other recent studies of the SCI population and found that female sex ratio, age at time of injury and survey were comparable to the global data, but ratio of complete injuries was higher, especially with complete paraplegia, in our study [19]. This was also true when compared with the domestic findings reporting lower rate of complete injuries [23] (41.2% vs. 58.8%). We can conclude that our study participants overrepresent the paraplegic complete subpopulation, and the representativeness of the study results is limited.

To address such issues and allow the analysis of non-responders and also enable a longitudinal comparison between the consequent waves of the InSCI survey, a fully identified, cohort database that represents the target population well and function as a sound sampling frame needs to be identified in the near future [19]. Consistent and stable data collection, in which the data could be compared on an individual basis, may make it possible to observe the incidence of new complications and disabilities. Furthermore, a population-based analysis will enable the evaluation of the impact of the recently introduced policies and practices as Ko-InSCI survey allows longitudinal data collection on the population. This would enable a more sophisticated analysis of the factors that contribute to the lives of individuals with SCI.

## 5. Conclusions

In conclusion, the Ko-InSCI study has added much understanding to the lives of people with disabilities in Korea and offers opportunities for further learning by enabling comparisons with studies from other countries with different health systems and social structures. As part of the LHS-SCI, health systems can learn from each other and evolve over time with consecutive waves of the surveys.

## Figures and Tables

**Table 1:** Number of participants based on the response modes.

Response Modes		*n*	Total
Self-administered	Online-based	331	886
	Paper-based	555	
Face-to-face interviewer-administered		4	4
Total		890	

**Table 2:** Sociodemographic and injury characteristics of the participants.

	Subgroup	N (%; 95% CI) or Median (IQR) ^1^
Age at time of survey (years)		48.00 (39.00–56.00)
Age at time of injury (years)		30.08 (22.21–39.83)
Time since injury (years)		14.67 (7.17–21.71)
Subjective social status		4.00 (2.00–6.00)
Sex		
	Female	214 (24.1; 21.4–27.1)
	Male	673 (75.9; 72.9–78.6)
Age group		
	18–30	87 (10.1; 8.3–12.3)
	31–45	268 (31.2; 28.2–34.4)
	46–60	398 (46.3; 43.0–49.7)
	61–75	101 (11.8; 9.8–14.1)
	76+	5 (0.6; 0.2–1.4)
Injury duration		
	~5	182 (21.2; 18.6–24.0)
	6–15	288 (33.5; 30.4–36.7)
	6–25	272 (31.6; 28.6–34.8)
	26+	118 (13.7; 11.6–16.2)
Injury level and extent		
	Complete paraplegia	348 (39.8; 36.6–43.1)
	Incomplete paraplegia	178 (20.4; 17.8–23.2)
	Complete tetraplegia	157 (18.0; 15.6–20.7)
	Incomplete tetraplegia	191 (219; 19.2–24.7)
SCI cause		
	Traumatic	815 (92.2; 90.2–93.8)
	Non-traumatic	69 (7.8; 6.2–9.8)
Paid work		
	No	628 (72.4; 69.4–75.3)
	Yes	239 (27.6; 24.7–30.6)
Household composition		
	Living alone	251 (28.2; 25.3–31.3)
	Living with kids and youths under 18	141 (15.8; 13.6–18.4)
	Living with adults from 18 to 64	480 (53.9; 50.6–57.2)
	Living with adults older than 64	160 (18.0; 15.6–20.6)
	Living in facility	9 (1.0; 0.5–1.9)
Highest education level		
	Primary	52 (5.9; 4.5–7.7)
	Lower-secondary	96 (10.9; 9.0–13.2)
	Higher-secondary	366 (41.7; 38.5–45.0)
	Post-secondary	24 (2.7; 1.8–4.0)
	Short tertiary	150 (17.1; 14.7–19.7)
	Bachelor or equivalent	145 (16.5; 14.2–19.1)
	Master or equivalent	45 (5.1; 3.8–6.8)
Household income		
	Less than 96	288 (33.8; 30.7–37.1)
	96–190	180 (21.2; 18.5–24.0)
	190–257	109 (12.8; 10.7–15.2)
	257–313	70 (8.2; 6.6–10.3)
	313–368	47 (5.5; 4.2–7.3)
	368–424	52 (6.1; 4.7–7.9)
	424–558	55 (6.5; 5.0–8.3)
	558–660	26 (3.1; 2.1–4.4)
	660–996	20 (2.4; 1.5–3.6)
	More than 996	4 (0.5; 0.2–1.2)
Partnership status		
	In partnership	432 (49.0; 45.7–52.3)
	Not in partnership	449 (51.0; 47.7–54.3)

^1^ IQR: Interquartile range.

**Table 3:** Differences in activity, participation, health conditions, quality of life, and environmental factors according to the age and sex.

Subgroup	m-SCIM-SR ^a^ Total Score		No. of A/P ^b^ Problems		NEFI-S ^c^ Score		No. of Health Conditions		Overall QOL ^d^	
Variable	Mean	Mean Diff (95% CI ^e^)	Mean	Mean Diff (95% CI)	Mean	Mean Diff (95% CI)	Mean	Mean Diff (95% CI)	Mean	Mean Diff (95% CI)
Sex										
Female	37.28	ref ^f^	8.58	ref	37.72	ref	9.68	ref	2.86	ref
Male	36.23	0.380 (−1.23–1.98)	8.80	−0.05 (−0.68–0.58)	36.66	−1.30 (−2.78–0.18)	10.32	0.43 (−0.13–1.00)	2.87	0.030 (−0.15–0.20)
*p*-value		0.644		0.879		0.087		0.133		0.745
Age group										
18–30	37.66	ref	7.53	ref	35.89	ref	8.52	ref	3.12	ref
31–45	38.11	1.30 (−1.18–3.77)	8.19	0.88 (-0.10–1.86)	36.20	0.73 (−1.55–3.01)	10.27	1.49 (0.62–2.36) **	2.96	−0.156 (−0.42–0.11)
46–60	35.93	−1.48 (−3.95–1.00)	9.03	1.63 (0.65–2.60) **	37.53	2.05 (−0.24–4.33)	10.46	1.57 (0.70–2.44) **	2.82	−0.232 (−0.50–0.04)
61–75	33.65	−2.96 (−5.97–0.05)	10.05	2.25 (1.06–3.43) **	37.06	1.25 (−1.52–4.03)	10.04	1.11 (0.05–2.17) *	2.62	−0.360 (−0.69–−0.03) *
76+	27.60	−4.85 (−13.72–4.02)	12.40	4.44 (0.94–7.94) *	42.80	7.28 (−0.91–15.47)	13.20	4.12 (1.00–7.24) *	2.60	−0.414 (−1.37–0.55)
*p*-value		0.002		<0.001		0.161		0.002		0.258

^a^. m-SCIM-SR: self-report version of modified Spinal Cord Independence Measure; ^b^. A/P: activity and participation; ^c^. NEFI-S: Nottwil Environmental Factors Inventory Short Form; ^d^. QOL: quality of life; ^e^. CI: confidence interval; ^f^. ref: reference; and * <0.05 ** <0.01.

**Table 4:** Differences in activity, participation, health conditions, quality of life, and environmental factors according to the lesion characteristics and paid work status.

Subgroup	m-SCIM-SR ^a^ Total Score		No. of A/P ^b^ Problems		NEFI-S ^c^ Score		No. of Health Conditions		Overall QOL ^d^	
Variable	Mean	Mean Diff ^f^ (95% CI ^e^)	Mean	Mean Diff (95% CI)	Mean	Mean Diff (95% CI)	Mean	Mean Diff (95% CI)	Mean	Mean Diff (95% CI)
Injury duration										
~5	36.94	ref ^f^	9.26	ref	37.82	ref	9.40	ref	2.83	ref
06–15	35.82	−1.12 (−3.06–0.82)	8.68	−0.62 (−1.38–0.15)	36.67	−1.15 (−2.94–0.64)	10.36	0.72 (0.04–1.40) *	2.90	0.04 (−0.17–0.25)
16–25	35.92	−1.82 (−3.82–0.18)	8.57	−0.83 (−1.62, −0.04) *	36.69	−1.33 (−3.18–0.51)	10.38	0.60 (−0.10–1.31)	2.85	0.02 (−0.20–0.23)
26+	38.68	−0.54 (−3.02–1.94)	8.56	−0.73 (−1.70–0.25)	36.69	−1.61 (−3.90–0.68)	10.32	0.70 (−0.17–1.57)	2.90	0.10 (−0.17–0.37)
*p*-value		0.310		0.215		0.451		0.194		0.897
Injury level and extent										
Complete paraplegia	37.66	ref	7.53	ref	35.89	ref	8.52	ref	3.12	ref
Incomplete paraplegia	38.11	1.30 (−1.18–3.77)	8.19	0.88 (−0.10–1.86)	36.20	0.73 (−1.55–3.01)	10.27	1.49 (0.62–2.36) **	2.96	−0.156 (−0.42–0.11)
Complete tetraplegia	35.93	−1.48 (−3.95–1.00)	9.03	1.63 (0.65–2.60) **	37.53	2.05 (−0.24–4.33)	10.46	1.57 (0.70–2.44) **	2.82	−0.232 (−0.50–0.04)
*p*-value		0.002		<0.001		0.161		0.002		0.258
SCI cause										
Traumatic	36.21	ref	8.77	ref	36.89	ref	10.22	ref	2.88	ref
Non-traumatic	39.90	0.75 (−1.93–3.42)	8.54	−0.38 (−1.43–0.68)	37.24	−0.19 (−2.66–2.28)	9.47	−0.57 (−1.51–0.38)	2.73	−0.08 (−0.37–0.22)
*p*-value		0.585		0.487		0.880		0.240		0.610
Paid work										
No	34.92	ref	9.27	ref	37.39	ref	10.34	ref	2.76	ref
Yes	40.55	4.68 (3.11–6.24) **	7.38	−1.40 (−2.02, 0.78) **	35.69	−1.20 (−2.65–0.24)	9.71	−0.57 (−1.12, −0.02) *	3.17	0.35 (0.18–0.52) **
*p*-value		<0.001		<0.001		0.103		0.044		<0.001

^a^. m-SCIM-SR: self-report version of modified Spinal Cord Independence Measure; ^b^. A/P: activity and participation; ^c^. NEFI-S: Nottwil Environmental Factors Inventory Short Form; ^d^. QOL: quality of life; ^e^. CI: confidence interval; ^f^. ref: reference; and * <0.05 ** <0.01.

## Data Availability

The datasets generated and analyzed during the current study are not publicly available. De-identified data may be made available upon request and with permission from the KoSCI committee.
